# Case Report A Rare Cause of Cardiofaciocutaneous Syndrome—KRAS p.Pro34arg: Clinical Delineation, Diagnostic Challenges, and Implications for RASopathy Classification

**DOI:** 10.3390/reports9040335

**Published:** 2026-10-05

**Authors:** Francisco J. Mérida De Ia Torre, Mónica Coll Vidal, Ivette Rubio Martínez, Lucía Quintana Tello, Ramón Brugada Terradellas

**Affiliations:** 1 Department of Genetic, Málaga Regional University Hospital, 29011 Málaga, Spain; 2 Department of Biochemistry, Molecular Biology and Immunology, University of Málaga, 29071 Málaga, Spain; 3 Gerona Institute of Biomedical Research (IDIBGI), 17190 Gerona, Spain; mcoll@gencardio.com; 4 Clinical Genetics Department, GENinCode SLU (Affiliate of GENinCode UK), 08244 Barcelona, Spain; irubio@genincode.com (I.R.M.); lquintana@genincode.com (L.Q.T.); 5 Department of Genetic, Gerona University Hospital Santa, Caterina, 17190 Gerona, Spain

**Keywords:** cardiofaciocutaneous syndrome, CFC2, *KRAS*, p.Pro34Arg, RASopathy, hypertrophic cardiomyopathy, ventriculomegaly, developmental delay, next-generation sequencing, genotype–phenotype correlation

## Abstract

**Background and Clinical Significance**: Cardiofaciocutaneous syndrome (CFC) is a rare autosomal dominant RASopathy characterized by craniofacial dysmorphism, congenital heart defects, cutaneous abnormalities, growth impairment, and neurodevelopmental delay. Pathogenic variants in *KRAS* account for a minority of cases and define cardiofaciocutaneous syndrome type 2 (CFC2). Establishing a molecular diagnosis is often challenging because of the substantial clinical overlap among RASopathies, including Noonan and Costello syndromes. **Case Presentation**: We describe a one-year-old boy presenting with macrocephaly, ventriculomegaly, hypotonia, developmental delay, hypertrophic cardiomyopathy, gastroesophageal reflux, and mild dermatological abnormalities. To clarify the diagnosis, a custom next-generation sequencing panel targeting RASopathy-associated genes was performed. Genetic analysis identified a heterozygous *KRAS* variant, NM_033360.4:c.101C>G, p.(Pro34Arg), which was confirmed by Sanger sequencing and shown to have arisen de novo through parental testing. Additional variants of uncertain significance were detected in *ABCC9*, *CACNA1G*, and *DES*; however, these were considered unlikely to account for the phenotype because of limited clinical concordance and current ACMG/AMP classification. The *KRAS* variant was classified as pathogenic based on its de novo occurrence, absence from population databases, localization within a critical functional domain, supportive computational evidence, and previously reported pathogenic substitutions affecting the same residue. **Conclusions**: This case provides additional evidence supporting the pathogenicity of *KRAS* p.(Pro34Arg), a variant reported only rarely in association with CFC. The observed phenotype further expands the clinical spectrum of KRAS-associated CFC and highlights the value of comprehensive molecular testing for the differential diagnosis of RASopathies and the refinement of genotype–phenotype correlations.

## 1. Introduction and Clinical Significance

RASopathies comprise a group of clinically and genetically heterogeneous developmental disorders caused by germline variants affecting components of the RAS/mitogen-activated protein kinase (RAS/MAPK) signaling pathway. Dysregulation of this signaling cascade results in a spectrum of overlapping phenotypes characterized by craniofacial dysmorphism, congenital heart disease, growth abnormalities, cutaneous manifestations, and neurodevelopmental impairment [1].

Cardiofaciocutaneous syndrome (CFC; OMIM #115150) is a rare autosomal dominant RASopathy first described in 1986 and characterized by distinctive craniofacial features, congenital cardiac defects, dermatologic abnormalities, feeding difficulties, developmental delay, and variable neurological involvement [1,2]. The diagnosis can be particularly challenging during infancy because of substantial phenotypic overlap with other RASopathies, especially Noonan syndrome (OMIM #163950) and Costello syndrome (OMIM #218040), making molecular testing essential for diagnostic confirmation [1,2].

The molecular basis of CFC is genetically heterogeneous. Pathogenic variants in *BRAF* account for approximately 75% of molecularly confirmed cases, whereas variants in *MAP2K1* and *MAP2K2* explain most of the remaining cases. Variants in *KRAS* are considerably less common and are associated with cardiofaciocutaneous syndrome type 2 (CFC2; OMIM #615278) [1,3]. Despite sharing a common pathogenic mechanism involving activation of the RAS/MAPK pathway, individuals carrying variants in different genes may exhibit variable neurological, cardiac, growth, and dermatological manifestations, reflecting the complexity of genotype–phenotype relationships within CFC [1,2,4].

Recent advances have further expanded the genetic landscape of CFC. In addition to the classical disease-associated genes, variants in *YWHAZ*, which encodes a member of the 14-3-3 protein family involved in RAF-MEK-ERK signaling regulation, have been reported in individuals with a cardiofaciocutaneous phenotype. Functional studies demonstrated increased RAF-ERK pathway activation, supporting the role of *YWHAZ* as a novel gene associated with CFC-like disease and emphasizing the central importance of RAS/MAPK dysregulation in the pathogenesis of these disorders [5].

Among the genes associated with CFC, *KRAS* represents one of the rarest causes of disease and accounts for only a small proportion of affected individuals [1,4]. The limited number of reported patients has restricted the establishment of robust genotype–phenotype correlations, particularly for individual variants. Consequently, detailed clinical descriptions of rare *KRAS* variants remain important for improving disease characterization and refining variant interpretation.

Here, we report a child presenting with macrocephaly, ventriculomegaly, hypotonia, developmental delay, hypertrophic cardiomyopathy, gastroesophageal reflux, and dermatologic manifestations in whom molecular testing identified the rare *KRAS* variant NM_033360.4:c.101C>G, p.(Pro34Arg). Given the extreme rarity of this variant and the limited phenotypic information currently available, this case provides additional evidence regarding its clinical significance and contributes to the phenotypic delineation of KRAS-associated CFC2.

## 2. Case Presentation

### 2.1. Clinical History

The proband was a one-year-old male of Arab ancestry and the youngest of three siblings born to non-consanguineous healthy parents. Pregnancy was uneventful, and delivery occurred at 38 weeks of gestation by vacuum-assisted vaginal delivery because of failure to progress. Birth weight was 3.4 kg, and Apgar scores were 9 and 10 at one and five minutes, respectively. No congenital abnormalities were identified during the neonatal period.

Following relocation to Spain, the patient required two hospital admissions during infancy. The first was due to a urinary tract infection that resolved with antibiotic treatment. The second admission was related to gastroesophageal reflux, which improved after dietary modification and formula change. Developmental concerns subsequently emerged, including delayed motor milestone acquisition and axial hypotonia, prompting neurological assessment.

### 2.2. Neuroimaging Findings

At seven months of age, the patient was referred to the Pediatric Neurosurgery Unit because of progressive macrocephaly associated with frontal prominence. Despite the increase in head circumference, he remained clinically stable, without vomiting, irritability, or signs of raised intracranial pressure. Physical examination revealed mild central hypotonia.

Computed tomography of the brain demonstrated ventricular enlargement compatible with hydrocephalus. Subsequent magnetic resonance imaging confirmed supratentorial ventriculomegaly with progressive ventricular dilatation, as reflected by an increase in Evans index from 0.46 to 0.54. Additional findings included aqueductal enlargement and bilateral frontal periventricular gliosis. These findings were considered consistent with evolving hydrocephalus.

### 2.3. Cardiac Evaluation

Comprehensive cardiological assessment was performed because congenital heart disease is a common component of RASopathies. Transthoracic echocardiography demonstrated concentric left ventricular hypertrophy with preserved systolic function. Mild tricuspid regurgitation was present, whereas pulmonary artery pressure remained within normal limits. No obstruction of the left ventricular outflow tract was detected. Aortic valve morphology and function were normal, and coronary artery origins were appropriately located. The pulmonary arteries, aortic arch, systemic venous return, and pulmonary venous return were structurally normal. No patent ductus arteriosus was identified. Electrocardiography showed sinus rhythm at 124 beats per minute, right-axis deviation, subtle prolongation of the PR interval, evidence of right ventricular enlargement, and positive T waves in the right precordial leads. The corrected QT interval was within normal limits.

### 2.4. Neurological and Developmental Assessment

Neurological follow-up evaluations were performed every six months. Physical examination demonstrated frontal bossing, scaphocephaly, low-set ears, and a mildly bulging anterior fontanelle. The overall craniofacial appearance was considered compatible with a cardiofaciocutaneous syndrome phenotype. Occipitofrontal circumference measured 47.5 cm (+0.6 SD).

Social interaction was appropriate for age, with normal response to auditory and visual stimuli. Babbling was present. Motor development was delayed, with inability to sit independently at the time of evaluation, although the patient could roll over and maintain a standing position with support. Neurological examination demonstrated preserved spontaneous limb movements, symmetric tendon reflexes, and flexor plantar responses. No clonus, focal neurological deficits, or abnormal involuntary movements were identified. Axial hypotonia was more pronounced than appendicular hypotonia. Metabolic screening investigations were negative.

The combination of developmental delay, central hypotonia, ventriculomegaly, macrocephaly, and characteristic craniofacial findings raised suspicion of an underlying syndromic disorder within the spectrum of RASopathies, prompting molecular genetic investigation.

### 2.5. Dermatological and Other Clinical Findings

Dermatological examination revealed mild cutaneous abnormalities characterized by dry skin and eczema-like manifestations. No severe hyperkeratosis, ichthyosis, extensive pigmentary abnormalities, or cutaneous hemangiomas were observed.

In addition to the neurological and cardiac manifestations, the patient exhibited gastroesophageal reflux during infancy, a feature commonly reported in cardiofaciocutaneous syndrome. No seizures, visual impairment, hearing deficits, or musculoskeletal deformities had been documented at the time of the present evaluation (Table 1).

## 3. Materials and Methods

### 3.1. Genetic Testing

Given the clinical suspicion of an underlying RASopathy based on the combination of macrocephaly, ventriculomegaly, developmental delay, central hypotonia, hypertrophic cardiomyopathy, gastroesophageal reflux, and dermatological abnormalities, genomic DNA was extracted from peripheral blood obtained from the proband.

Targeted next-generation sequencing (NGS) was performed using a custom gene panel designed for the evaluation of congenital hydrocephalus, neurodevelopmental disorders, hypotonia, congenital heart disease, and syndromic conditions associated with dysregulation of the RAS/MAPK signaling pathway. Library preparation and target enrichment were performed according to the manufacturer’s recommendations. Sequencing was carried out on an Illumina MiSeq platform (Illumina, San Diego, CA, USA).

Sequence reads were aligned against the human reference genome GRCh38/hg38. Variant calling, annotation, and prioritization were performed using a validated bioinformatic pipeline. The mean sequencing depth was approximately 300×, and more than 99% of target bases achieved a minimum coverage of 20×.

### 3.2. Variant Filtering and Interpretation

Variant prioritization focused on rare coding and splice-site variants potentially relevant to the patient’s phenotype. Variants with a minor allele frequency greater than 1% in population databases, including gnomAD, were excluded from further analysis. The remaining variants were assessed according to phenotype concordance, inheritance pattern, published literature, ClinVar classifications, population frequency databases, and in silico prediction tools.

Variant interpretation and classification were performed according to the recommendations of the American College of Medical Genetics and Genomics and the Association for Molecular Pathology (ACMG/AMP) [6].

Analysis identified a heterozygous *KRAS* variant, NM_033360.4: c.101C>G, p.(Pro34Arg), together with three variants of uncertain significance in *ABCC9* NM_005691: c.1987C>T, p.(Arg663Cys) (PP3, PM2), *CACNA1G* NM_018896: c.3990G>A, p.(Val1330=) (BP7, BA1) and *DES* NM_001927: c.458A>G, p.(Glu153Gly) (PP3, PM2). These additional variants were considered unlikely to explain the patient’s phenotype because the disorders currently associated with these genes do not adequately account for the combination of ventriculomegaly, developmental delay, central hypotonia, hypertrophic cardiomyopathy, craniofacial dysmorphism, and dermatological manifestations observed in the present case.

The *KRAS* p.(Pro34Arg) variant was classified as pathogenic according to ACMG/AMP guidelines [6]. Evidence supporting pathogenicity included confirmed de novo occurrence in a patient with a phenotype highly specific for a RASopathy (PS2), localization within a critical functional domain of the KRAS protein (PM1), absence from population databases (PM2), computational evidence supporting a deleterious effect, including a REVEL score of 0.905 (PP3), and the presence of previously reported pathogenic missense substitutions affecting the same amino acid residue (PM5) [7,8,9].

### 3.3. Sanger Validation and Segregation Analysis

The *KRAS* NM_033360.4:c.101C>G (p.Pro34Arg) variant identified by NGS was independently confirmed by bidirectional Sanger sequencing.

Segregation analysis was subsequently performed in both parents using genomic DNA extracted from peripheral blood samples. The variant was absent in both parental samples, confirming its de novo occurrence in the proband.

Representative electropherograms from the proband and both parents are provided in Supplementary Figure S1. The proband demonstrated the heterozygous C>G substitution, whereas both parents showed the wild-type sequence at the corresponding nucleotide position.

### 3.4. Ethical Considerations

This article describes a single clinical case and does not constitute biomedical research involving human subjects. According to current Spanish legislation governing routine clinical practice and observational case reports, approval by an Institutional Review Board was not required.

Written informed consent for genetic testing and publication of anonymized clinical and genetic information was obtained from the patient’s legal guardians prior to manuscript preparation.

All procedures were conducted in accordance with the principles of the Declaration of Helsinki.

## 4. Discussion

Cardiofaciocutaneous syndrome (CFC) is a rare developmental disorder belonging to the group of RASopathies, which result from dysregulation of the RAS/MAPK signaling pathway. Pathogenic variants affecting components of this pathway lead to disturbances in cell proliferation, differentiation, and development, producing a spectrum of overlapping phenotypes characterized by craniofacial dysmorphism, congenital heart disease, dermatological abnormalities, growth impairment, and neurodevelopmental delay [1,2]. The genetic landscape of CFC is heterogeneous and includes variants in *BRAF*, *MAP2K1*, *MAP2K2*, and, less frequently, *KRAS*, which is associated with cardiofaciocutaneous syndrome type 2 (CFC2; OMIM #615278) [1,3,4].

More recently, variants in *YWHAZ*, a member of the 14-3-3 protein family involved in RAF-MEK-ERK signaling regulation, have been proposed as an additional cause of a cardiofaciocutaneous phenotype, further expanding the molecular spectrum of CFC and highlighting the central role of RAS/MAPK pathway activation in disease pathogenesis [5].

The present patient exhibited many of the characteristic manifestations of CFC, including craniofacial dysmorphism, developmental delay, central hypotonia, ventriculomegaly, gastroesophageal reflux, dermatological abnormalities, and hypertrophic cardiomyopathy. This constellation of findings is highly consistent with the phenotypic spectrum described in previous clinical series and management guidelines for CFC [1,2,4].

The differential diagnosis among RASopathies remains particularly challenging during infancy because substantial phenotypic overlap exists between CFC, Noonan syndrome, and Costello syndrome. In the present case, the coexistence of significant neurological involvement, hypertrophic cardiomyopathy, characteristic facial features, and cutaneous abnormalities suggested a diagnosis within the CFC spectrum. Molecular testing was therefore essential for establishing a definitive diagnosis and excluding other clinically overlapping RASopathies [1,2].

A major finding of this report is the identification of the rare *KRAS* variant NM_033360.4:c.101C>G, p.(Pro34Arg). Germline *KRAS* variants account for only a small proportion of individuals with CFC and define CFC2, one of the least common molecular subtypes of the disorder [1,3]. Since the original report by Niihori et al., relatively few patients harboring pathogenic *KRAS* variants have been described, limiting the establishment of robust genotype–phenotype correlations and making detailed case descriptions particularly valuable [7].

Pro34 is located within the KRAS effector region (residues 32–40), a highly conserved domain responsible for interactions with downstream signaling effectors, including RAF family proteins (Figure 1) [7]. This region plays a critical role in mediating downstream RAS/MAPK pathway signaling. The substitution of a rigid proline residue by arginine may alter local conformational properties and effector binding, potentially affecting signal transduction. Although no functional studies specific to p.(Pro34Arg) are currently available, its localization within a critical functional domain provides a biologically plausible mechanism supporting pathogenicity. Furthermore, additional pathogenic substitutions affecting the same residue, including p.(Pro34Gln) and p.(Pro34Leu), have been reported in germline KRAS-related disorders [8,9], further reinforcing the functional importance of codon 34. Published pathogenic *KRAS* variants associated with CFC and related phenotypes are summarized in Table 2.

To our knowledge, p.(Pro34Arg) has previously been reported in only one individual presenting with a cardiofaciocutaneous phenotype [7]. Although the very limited number of reported patients precludes definitive conclusions regarding variant-specific manifestations, several clinical similarities can be identified between the previously reported patient and the present case. Both individuals exhibited developmental delay, hypotonia, neurological abnormalities, and cardiac involvement, supporting the pathogenic role of this variant in CFC2 [7].

Interestingly, dermatological manifestations in our patient were relatively mild and consisted primarily of dry skin and eczema-like lesions. Niihori et al. observed that individuals with *KRAS*-related CFC appeared to show less pronounced cutaneous abnormalities than those carrying *BRAF* variants, particularly regarding ichthyosis, hyperkeratosis, and hemangiomas [7]. Although the number of reported patients remains too small to establish reliable genotype–phenotype correlations, the cutaneous findings observed in the present case are compatible with this observation and contribute additional information regarding the phenotypic variability of KRAS-associated CFC.

The molecular findings further strengthen the diagnosis. According to ACMG/AMP recommendations, the identified variant fulfills multiple criteria supporting pathogenicity, including confirmed de novo occurrence, absence from population databases, localization within a critical functional domain, supporting computational evidence, and previously reported pathogenic substitutions affecting the same amino acid residue [6]. Together, these data provide strong evidence that *KRAS* p.(Pro34Arg) represents the principal molecular cause of the phenotype observed in this patient.

Several limitations should nevertheless be acknowledged. First, conclusions regarding genotype–phenotype associations remain inherently limited because only a very small number of individuals carrying the p.(Pro34Arg) variant have been reported. Second, the genetic analysis was performed using a targeted next-generation sequencing panel focused on genes associated with RASopathies. Although this approach enabled identification of the causative KRAS variant, broader genomic strategies such as whole-exome sequencing or whole-genome sequencing might have detected additional variants of potential clinical relevance. Third, although additional variants of uncertain significance were identified in ABCC9, CACNA1G, and DES, current evidence does not support these variants as the primary explanation for the patient’s clinical presentation. Finally, additional patients and functional studies will be necessary to better define the biological consequences of codon 34 substitutions and determine whether specific clinical characteristics are consistently associated with this variant.

Although the clinical presentation is most parsimoniously explained by the de novo *KRAS* p.(Pro34Arg) variant, the potential contribution of additional genetic factors should be considered. The targeted NGS panel also identified variants of uncertain significance in *ABCC9*, *CACNA1G*, and *DES*. None of the identified VUS has previously been associated with hydrocephalus, cardiofaciocutaneous syndrome, or RAS/MAPK pathway dysregulation, further reducing the likelihood of a significant contribution to the primary phenotype. Furthermore, the clinical manifestations typically associated with disorders involving these genes do not adequately account for the combination of ventriculomegaly, craniofacial dysmorphism, developmental delay, hypotonia, hypertrophic cardiomyopathy, and dermatological abnormalities observed in our patient. For these reasons, an oligogenic model was considered but judged unlikely. Nevertheless, given the increasing recognition of genetic modifiers and complex inheritance mechanisms in neurodevelopmental disorders, a subtle contribution of these additional variants to phenotypic variability cannot be completely excluded. Further functional studies and the identification of additional patients carrying similar variant combinations will be necessary to clarify any potential modifying effects.

Overall, the present report expands the clinical characterization of CFC2 associated with *KRAS* p.(Pro34Arg) and provides additional evidence supporting its pathogenicity. Continued reporting of rare *KRAS* variants will be essential for improving clinical interpretation, refining genotype–phenotype correlations, and enhancing our understanding of the molecular and phenotypic diversity of cardiofaciocutaneous syndrome [1,4].

## 5. Conclusions

Cardiofaciocutaneous syndrome (CFC) remains a diagnostically challenging RASopathy because of its significant phenotypic overlap with other disorders involving dysregulation of the RAS/MAPK signaling pathway [1,2].

In the present patient, the combination of craniofacial dysmorphism, developmental delay, ventriculomegaly, central hypotonia, hypertrophic cardiomyopathy, gastroesophageal reflux, and dermatological abnormalities raised clinical suspicion of CFC, which was subsequently confirmed through molecular genetic testing.

The identification of the rare *KRAS* NM_033360.4:c.101C>G, p.(Pro34Arg) variant, classified as pathogenic according to ACMG/AMP criteria [6], provides additional evidence supporting its role in cardiofaciocutaneous syndrome type 2 (CFC2) [3]. Given that this variant has been reported only once previously in association with a cardiofaciocutaneous phenotype, the present report contributes valuable clinical information regarding its phenotypic presentation [7].

Although robust genotype–phenotype correlations cannot be established from such a limited number of observations, the overlap between the previously reported patient and the present case further supports the clinical relevance of *KRAS* p.(Pro34Arg) in CFC2 [7]. Future reports and functional studies will be required to better define the phenotypic spectrum associated with this rare variant and to clarify potential variant-specific manifestations [1,4].

Finally, this case highlights the importance of integrating detailed clinical assessment with comprehensive molecular testing in children presenting with complex neurodevelopmental and cardiovascular manifestations suggestive of a RASopathy [1,2]. Continued reporting of patients harboring rare *KRAS* variants will be essential to improve variant interpretation, refine clinical characterization, and expand current knowledge of KRAS-associated CFC2 [3,4,7].

## Figures and Tables

**Table 1:** Comparison of major RASopathies using HPO terminology.

Feature (HPO)	Noonan Syndrome	Costello Syndrome	CFC-BRAF	CFC-KRAS	Present Patient
Macrocephaly (HP:0000256)	Variable	Occasional	Common	Common	Present
Hypertrophic cardiomyopathy (HP:0001639)	Occasional	Frequent	Frequent	Frequent	Present
Pulmonary stenosis (HP:0001642)	Common	Less common	Variable	Variable	Absent
Ventriculomegaly (HP:0002119)	Rare	Rare	Frequent	Frequent	Present
Hypotonia (HP:0001252)	Common	Common	Common	Common	Present
Developmental delay (HP:0001263)	Variable	Common	Common	Common	Present
Dry skin (HP:0000958)	Variable	Variable	Frequent	Variable	Present
Ichthyosis (HP:0008064)	Rare	Rare	Frequent	Rare	Absent
Hyperkeratosis (HP:0000962)	Rare	Rare	Frequent	Rare	Absent

**Table 2:** Published KRAS variants associated with cardiofaciocutaneous syndrome.

p.Pro34Arg	CFC2	De novo	Ventriculomegaly, cardiomyopathy, developmental delay
p.Pro34Leu	CFC2	De novo	Developmental delay, distinctive facial features
p.Pro34Gln	CFC2	De novo	Cardiac involvement, neurodevelopmental delay
p.Lys5Glu	CFC2	De novo	Severe developmental impairment
p.Gly60Arg	CFC2	De novo	Typical CFC phenotype
p.Asp153Val	CFC2	De novo	Cardiac and neurological manifestations

## Data Availability

The data presented in this study are available on request from the corresponding author. The data are not publicly available due to privacy restrictions.

## Supplementary Materials

The following supporting information can be downloaded at: https://www.mdpi.com/article/10.3390/reports9040335/s1. Table S1. Genes included in the custom panel; Figure S1. Representative electropherograms from the proband and both parents.
