# Supplementary material for: Case Report A Rare Cause of Cardiofaciocutaneous Syndrome—KRAS p.Pro34arg: Clinical Delineation, Diagnostic Challenges, and Implications for RASopathy Classification
Source: Reports (MDPI). 2026 Oct 5;9(4):335. doi: 10.3390/reports9040335 (PMC13692532; doi:10.3390/reports9040335)

Table S1. Genes included in the custom panel

ABCC9, ACTA2, ACTC1, ACTN2, AKAP9, ANK2, ANKRD1, BAG3, BRAF, CACNA1C, CACNA1G, CACNA1H, CACNA1I, CACNA2D1, CACNB2, CALM1, CALM2, CALM3, CALR3, CASQ2, CAV3, CBL, COL3A1, CRYAB, CSRP3, CTF1, CTNNA3, DES, DMD, DMPK, DPP6, DSC2, DSG2, DSP, DTNA, ECE1, EMD, EN1, EYA1, EYA4, FBN1, FBN2, FHL2, FKTN, FLNA, FLNC, GAA, GJA1, GJA5, GLA, GPD1L, HCN1, HCN2, HCN4, HRAS, JPH2, JUP, KCNA5, KCND3, KCNE1, KCNE2, KCNE3, KCNE4, KCNE5, KCNH2, KCNJ2, KCNJ5, KCNJ8, KCNQ1, KRAS, LAMA4, LAMP2, LDB3, LMNA, MAP2K1, MAP2K2, MYBPC3, MYH11, MYH6, MYH7, MYL2, MYL3, MYLK2, MYOZ2, MYPN, NEBL, NEXN, NF1, NOS1AP, NOTCH1, NPPA, NRAS, NUP155, PDLIM3, PHOX2A, PHOX2B, PITX2, PKP2, PLN, PRKAG2, PTPN11, RAF1, RANGRF, RBM20, RET, RIT1, RYR2, SCN10A, SCN1B, SCN2B, SCN3B, SCN4B, SCN5A, SDHA, SGCD, SHOC2, SLC22A5, SLC6A4, SLC8A1, SLMAP, SLN, SMAD3, SNTA1, SOS1, SOS2, SPRED1, TAZ, TCAP, TGFB2, TGFB3, TGFBR1, TGFBR2, TGFBR3, TLX3, TMEM43, TMPO, TNNC1, TNNI3, TNNT2, TP63, TPM1, TRDN, TRIM63, TRPM4, TTN, TTR, VCL

Figure S1. Representative electropherograms from the proband and both parents.

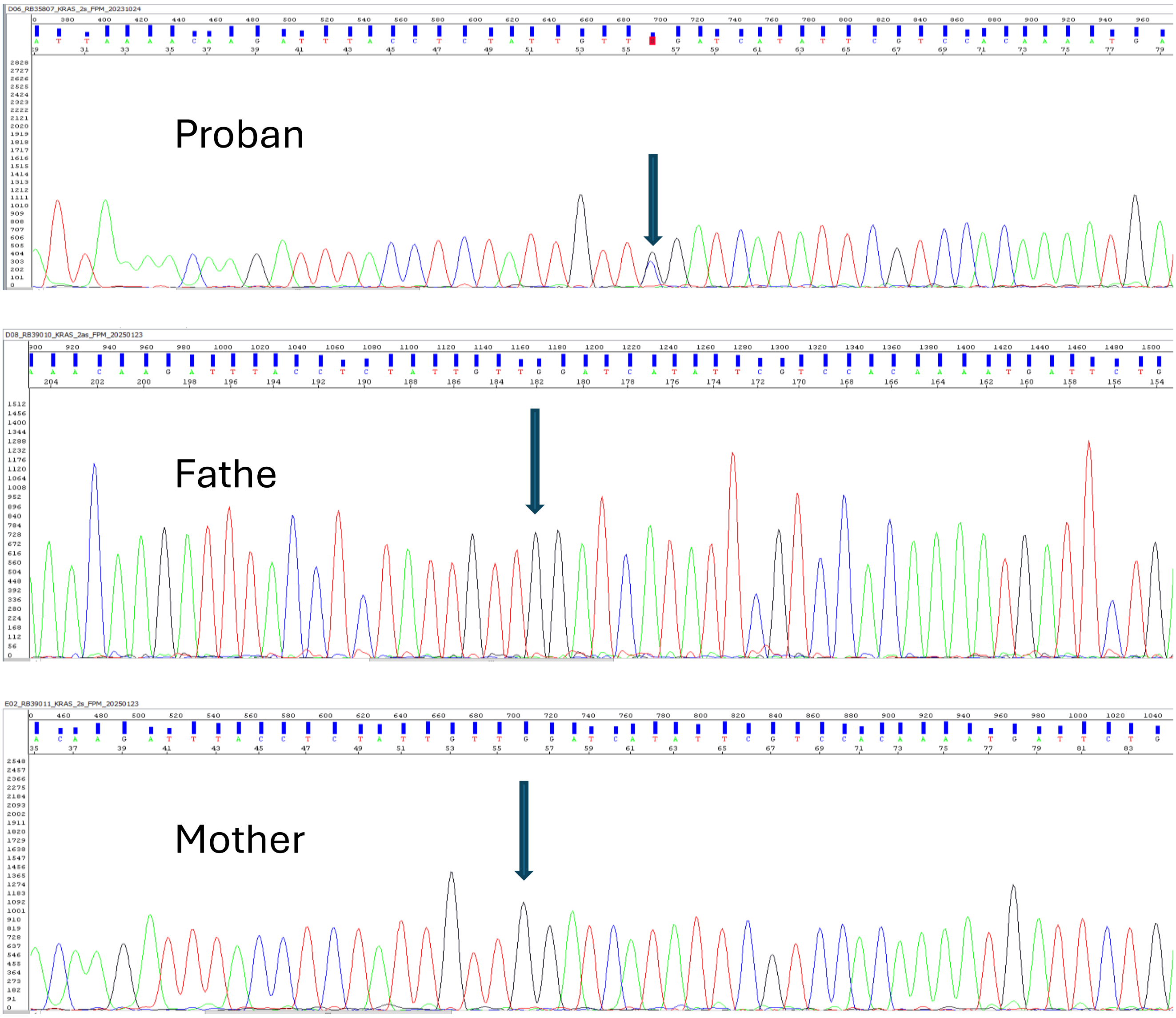

Supplement: Supplementary file 1 [file reports-09-00335-s001.zip › reports-4554147-supplementary.pdf]
